# Alternative splicing of Apoptosis Stimulating Protein of TP53-2 (ASPP2) results in an oncogenic isoform promoting migration and therapy resistance in soft tissue sarcoma (STS)

**DOI:** 10.1186/s12885-022-09726-7

**Published:** 2022-07-02

**Authors:** Vasileia Tsintari, Bianca Walter, Falko Fend, Mathis Overkamp, Christian Rothermundt, Charles D. Lopez, Marcus M. Schittenhelm, Kerstin M. Kampa-Schittenhelm

**Affiliations:** 1 Department of Hematology, Oncology, Clinical Immunology and Rheumatology, University Hospital Tübingen (UKT), Tübingen, Germany; 2 Institute of Pathology, University Hospital Tübingen, Tübingen, Germany; 3 Department of Medical Oncology and Hematology, Cantonal Hospital St. Gallen (KSSG), St. Gallen, Switzerland; 4 Department of Hematology and Medical Oncology, Oregon Health and Science University (OHSU), Portland, OR USA; 5 Laboratory of Translational Experimental Hematology and Oncology, Medical Research Center and Department of Medical Oncology and Hematology, Cantonal Hospital, Rorschacherstr. 95, St. Gallen, 9007 Switzerland St.Gallen; 6 St. Gallen, Switzerland

**Keywords:** Soft tissue sarcoma, Rhabdomyosarcoma, Alternative splicing, ASPP2κ, p53, Oncogenes, Tumor suppressor, Apoptosis, Therapy resistance

## Abstract

**Background:**

Metastatic soft tissue sarcoma (STS) are a heterogeneous group of malignancies which are not curable with chemotherapy alone. Therefore, understanding the molecular mechanisms of sarcomagenesis and therapy resistance remains a critical clinical need. ASPP2 is a tumor suppressor, that functions through both p53-dependent and p53-independent mechanisms. We recently described a dominant-negative ASPP2 isoform (*ASPP2κ)*, that is overexpressed in human leukemias to promote therapy resistance. However, ASPP2κ  has never been studied in STS.

**Materials and methods:**

Expression of ASPP2κ was quantified in human rhabdomyosarcoma tumors using immunohistochemistry and qRT-PCR from formalin-fixed paraffin-embedded (FFPE) and snap-frozen tissue. To study the functional role of ASPP2κ in rhabdomyosarcoma, isogenic cell lines were generated by lentiviral transduction with short RNA hairpins to silence ASPP2κ expression. These engineered cell lines were used to assess the consequences of ASPP2κ silencing on cellular proliferation, migration and sensitivity to damage-induced apoptosis. Statistical analyses were performed using Student’s t-test and 2-way ANOVA.

**Results:**

We found elevated *ASPP2κ* mRNA in different soft tissue sarcoma cell lines, representing five different sarcoma sub-entities. We found that *ASSP2κ* mRNA expression levels were induced in these cell lines by cell-stress. Importantly, we found that the median *ASPP2κ* expression level was higher in human rhabdomyosarcoma in comparison to a pool of tumor-free tissue. Moreover, *ASPP2κ* levels were elevated in patient tumor samples versus adjacent tumor-free tissue within individual patients. Using isogenic cell line models with silenced ASPP2κ expression, we found that suppression of ASPP2κ enhanced chemotherapy-induced apoptosis and attenuated cellular proliferation.

**Conclusion:**

Detection of oncogenic *ASPP2κ* in human sarcoma provides new insights into sarcoma tumor biology. Our data supports the notion that ASPP2κ promotes sarcomagenesis and resistance to therapy. These observations provide the rationale for further evaluation of ASPP2κ as an oncogenic driver as well as a prognostic tool and potential therapeutic target in STS.

**Supplementary Information:**

The online version contains supplementary material available at 10.1186/s12885-022-09726-7.

## Background

Soft tissue sarcoma (STS) are a rare and heterogeneous group of malignancies of mesenchymal origin, accounting for less than 1% of all human malignancies, which comprises an annual incidence of 30/million [1, 2]. According to the revised 2020 WHO classification, sarcomas are classified into more than 100 histological subtypes [3] arising from muscle, fat, or deep skin tissue but also joints, nerves or blood vessels.

Treatment options in advanced STS are still not satisfying for most entities. Standard chemotherapy in non-resectable STS is based on anthracyclines, but efficacy rates are rather moderate and patients ultimately relapse and die of the disease.

The Apoptosis Stimulating Proteins of TP53 (ASPP) represent a family of key apoptosis regulators within the TP53 pathway and consist of two pro-apoptotic (ASPP1 and ASPP2) and one anti-apoptotic member (iASPP) [4]. All three share an evolutionarily conserved C-terminus that includes four ankyrin repeats, an SH3-domain and a proline-rich region, which directly interacts with the TP53 core domain (ASPP1/2) or an adjacent linker region (iASPP) to increase or inhibit the affinity of TP53 to promoters of proapoptotic genes [5–7].

Attenuation of the ASPP2 wildtype isoforms is frequently observed in various tumors such as breast cancer [6], high-grade lymphoma [8] and acute leukemia [9], where low ASPP2 expression levels are associated with a more aggressive disease, therapy failure, and poor clinical outcome. Furthermore, two mouse models have shown that ASPP2 is an independent haploinsufficient tumor suppressor, which shares common functions with TP53 [6, 10, 11]. While Aspp2^(−/−)^ mice were not viable, hemizygous (+/-) mice appeared developmentally normal but presented with an accelerated cellular proliferation rate in mouse embryonic fibroblasts (MEF) [9, 12] and an increased incidence of spontaneous tumors – especially lymphoma and sarcoma entities [10].

Importantly, we have recently described a novel stress-inducible splicing variant of *ASPP2*, named *ASPP2κ*, with a high prevalence in acute leukemia [13]. Exon-skipping results in a reading-frame shift with a premature translation stop, omitting most of the C-terminus, which harbors the TP53-binding sites. Consequently, direct interaction of the truncated *ASPP2κ* isoform and TP53 is predicted to be abrogated (similar to the situation in *TP53*-mutated cancers, where *mut-TP53* lacks the ASPP2 binding sites [14]). ASPP2κ displays dominant-negative functions, which include increased proliferation rates along with impaired induction of apoptosis pathways. The functional consequences of ASPP2κ are thereby similar to a loss of the ASPP2 wildtype isoform, posing a risk to trigger early oncogenesis as well as impairing the response to DNA-damaging cancer therapeutics [13].

Preliminary data suggest that ASPP2κ is expressed in other tumor entities beyond leukemia as well [13]. However, the distribution and the functional role of ASPP2κ remain unknown. We therefore now expanded our studies to other neoplasms of mesenchymal origin and demonstrate frequent expression of the dominant-negative ASPP2κ-isoform in soft tissue sarcoma (STS), especially in rhabdomyosarcoma. Further, we demonstrate that ASPP2κ is an important factor in the biology of sarcoma, affecting tumor cell proliferation, and apoptosis, proposing a resistance mechanism towards anthracycline-based chemotherapy. Tantalizingly, a so far unknown functional mechanism in cellular migration is described, arguing for a role of ASPP2κ in metastasis.

Detection of oncogenic *ASPP2κ* in human sarcoma supports the notion that ASPP2κ promotes sarcomagenesis and resistance to therapy. Our findings provide the proof-of-concept for further evaluation of ASPP2κ as an oncogenic driver to define tumors at risk to metastasize, as well as a prognostic tool and potential therapeutic target in human STS.

## Methods

### Patient tissue collection

Patient rhabdomyosarcoma (Supplemental Table 1) and liposarcoma tissue (Supplemental Table 2), (formalin-fixed paraffin-embedded (FFPE) and snap-frozen tissue) and clinical data from consented patients were obtained from the central Biobank of the Comprehensive Cancer Centre Tübingen-Stuttgart after approval by the local ethics committee (188/2018BO2). Microscopically tumor-free tissue, obtained from adjacent tumor-surrounding areas from rhabdomyosarcoma patients served as controls.

### Cell lines

Soft tissue sarcoma (STS) cell lines (SK-LMS, SW982, RD, SW872) as well as primary sarcoma cell lines (ssRMS, BR-CS and WW-LMS) isolated from consented rhabdomyosarcoma patients` primary tumors, were a gift of Dr. med. C. Hinterleitner and Prof. G. Kopp (University of Tübingen).

Cell lines SK-LMS, SW982, RD, ssRMS, BR-CS, and WW-LMS were maintained in Dulbecco’s Minimum Essential Media (DMEM, Gibco) supplemented with 10% fetal bovine serum (FBS) (Sigma-Aldrich), 1% penicillin–streptomycin (Biochrom), 1% Sodium pyruvate and 1% MEM-Non-Essential Amino acids (100X) (Gibco), while the SW872 was maintained in RPMI supplemented with 10% fetal bovine serum (FBS) (Sigma-Aldrich), 1% penicillin–streptomycin (Biochrom), 1% Sodium pyruvate and 1% MEM-Non-Essential Amino acids (100X) (Gibco).

HEK239T cells used for lentiviral pseudo-virus production were obtained from ThermoFisher Scientific and maintained in Hyclone-DMEM medium supplemented with 10% FBS and 200 µM L-glutamine.

All cell lines were cultivated at 37 °C in 5% CO2 humidity.

### RNA extraction, cDNA synthesis, and qRT-PCR

mRNA extracted from fresh frozen tissue or tumor cell lines was isolated using the RNeasy® RNA purification kit (Qiagen) – and cDNA was synthesized using the Reverse Transcriptase Kit from Roche.

Quantitative real-time PCR analysis was performed on a qRT-PCR Roche® LightCycler in triplicates, using the Light Cycler 480 Probes Master (Roche). Relative quantification of the target gene transcript in comparison to a reference transcript was calculated using the Cp method. Isoform-specific primers for *ASPP2κ*, specifically targeting the unique sequence of the splicing junction, were custom made (Eurofins). GAPDH was used as a housekeeping gene reference control.

### ASPP2κ protein expression in FFPE patient tissue

A BenchMark ULTRA fully automated staining instrument (Roche) loaded with a custom-made polyclonal anti-ASPP2κ antibody [13] was used to determine ASPP2κ protein expression levels in a panel of 11 native rhabdomyosarcoma samples. Slides were assessed using the OptiView DAB Immunohistochemistry Detection kit (Roche).

### Lentiviral *ASPP2κ*-interference

Recombinant lentiviruses, expressing a custom-made short hairpin (sh) RNA against *ASPP2κ* were produced according to the provider’s guidelines. Briefly, a pre-selected pGFP-C-shLenti vector (Origene) was custom designed containing an shRNA expression cassette against *ASPP2κ*. A trans-lentiviral packaging kit (Dharmacon) was used to generate replication-incompetent lentiviral particles in HEK293T producer cells. Viral particles were stored at -80 °C for further use.

Two sarcoma cell lines, RD and ssRMS, were used to establish stable Isoform-specific *ASPP2κ* knockdown strains. Empty vector (EV) strains were developed as negative controls. After lentiviral transduction and puromycin selection, transduction efficiency was evaluated by analysis of GFP expression. Cells were further kept in medium containing a low puromycin concentration (0,2 μg/ml).

### Proliferation assay

Cell doubling times were assessed daily, using a hemocytometer after trypan blue staining to compare the proliferation characteristics of *ASPP2κ*-interferenced cell models compared to the control cell strains. Experiments were performed in technical triplicates.

### Apoptosis assay

An annexin V-based protocol was used as previously described [13]. In short, dose dilution experiments were set up, using doxorubicin dissolved in DMSO. Cells were cultured for 48 h and stained with annexin V and 7-AAD to assess the proportion of apoptotic cells on a FACS Calibur (Becton Dickinson) flow cytometer. Experiments were performed in technical triplicates. DMSO carrier controls were performed accordingly.

### Migration assay (wound healing assay)

To determine and compare the migration capacity of ASPP2κ*-*interference cell models, a wound healing migration assay was performed: Cells were seeded and grown to a 90–95% confluent monolayer and scraped to produce a linear ‘wound’, using sterile 20 μl pipette tips. Migration of cells into the wound area was followed over time using a photomicroscope loaded with NIS Elements software (Nikon) at 10X magnification. Wound healing was quantified using TScratch software (www.cse-lab.ethz.ch) [15]. Experiments were performed in technical triplicates.

### Statistical analysis

All statistical analyses were carried out using Prism software (GraphPad). Quantitative variables were analyzed by Student’s t-test (paired and unpaired) or 2-way ANOVA as indicated. All statistical analyses were two-sided, and *p* < 0.05 was considered statistically significant.

## Results

### Detection of ASPP2κ in sarcoma cell lines

**Fig. 1:**
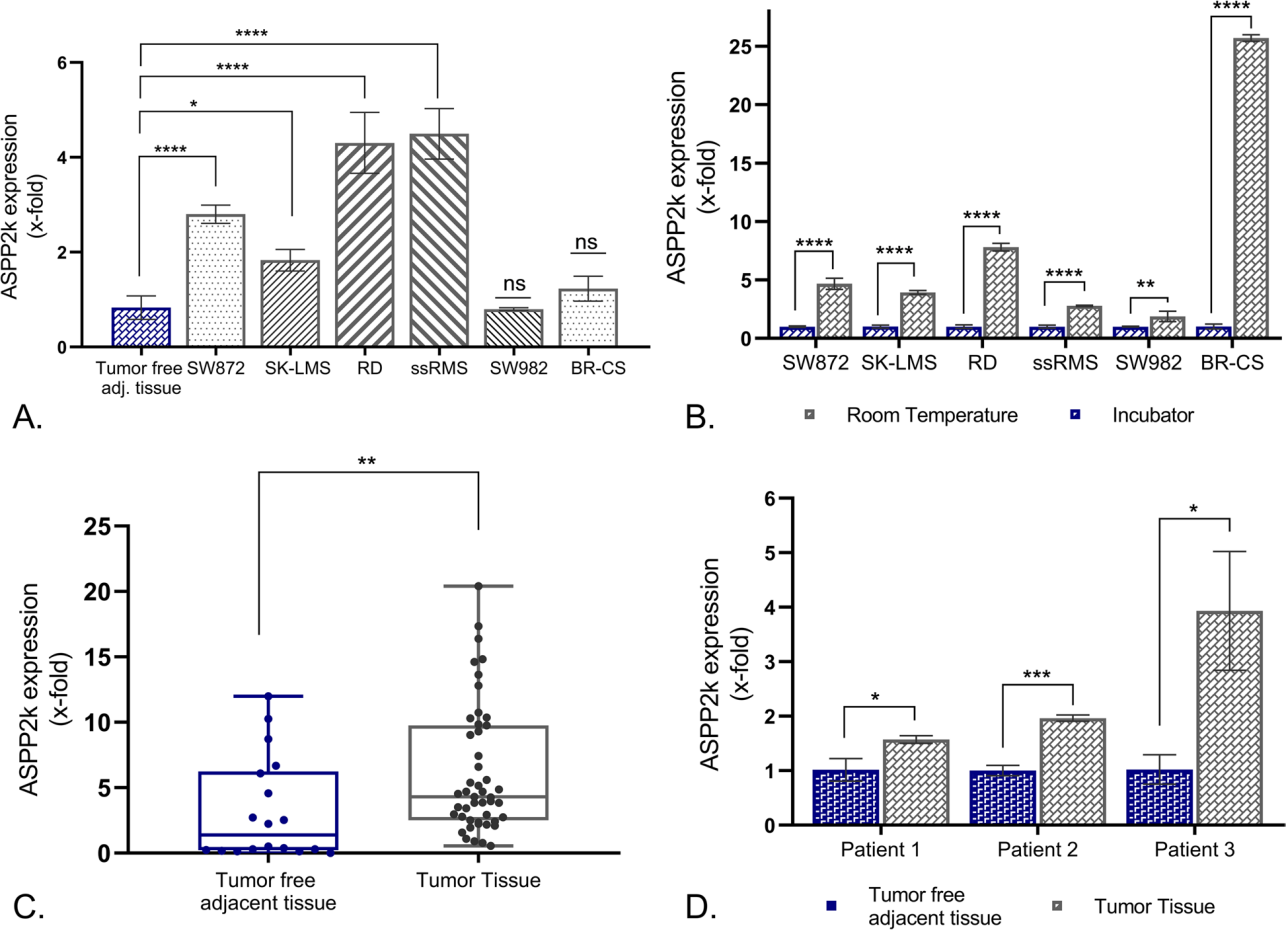
**A** Isoform-specific qRT-PCR of *ASPP2κ* in STS cell lines. A pool of microscopically tumor-free tissue (*n* = 15) deriving from STS patient samples (6 rhabdomyosarcoma, 9 liposarcoma) served as control. Statistical test: one-way ANOVA **B** Stress inducible, temperature-dependent expression levels of *ASPPκ* in STS cell lines as assessed by isoform-specific qRT-PCR. Statistical test: two-way ANOVA. **C** *ASPP2κ*-specific qRT-PCR assay to determine relative mRNA expression levels in rhabdomyosarcoma patient tissue (*n* = 15). A pool (*n* = 6) of tumor-free tissue derived from the same patients served as control. Patient samples were measured in technical triplicates. Statistical tests: unpaired t-test. **D** *ASPP2κ-*specific qRT-PCR assay to determine relative mRNA expression levels of tumor vs. tumor-free tissue from same individuals (*n* = 3). Statistical test: two-way ANOVA. ****p < 0.0001, ***p < 0.001, ** *p* < 0.01, *p < 0.05, ns (not significant); GAPDH served as housekeeping gene

To evaluate whether ASPP2κ is expressed in STS, we first analyzed *ASPP2κ* mRNA expression levels using an isoform-specific qRT-PCR in 6 different soft tissue sarcoma cell lines, representing five different sarcoma sub-entities (i.e., liposarcoma: SW872, leiomyosarcoma: SK-LMS, rhabdomyosarcoma: RD, spindle cell/sclerosing rhabdomyosarcoma: ssRMS, synovial sarcoma: SW982 and chondrosarcoma: BR-CS). Interestingly, four out of the six tested cell lines showed statistically significantly elevated *ASPP2κ* expression levels in comparison to the expression levels of pooled, adjacent, tumor-free tissue, derived from rhabdomyosarcoma (6) and liposarcoma (9) excidates (Fig. 1A).

### ASPP2κ is stress-inducible in sarcoma

We recently provided evidence in a leukemia model that ASPP2κ is stress-inducible, e.g., by chemotherapy, temperature, or radiation [13]. To confirm this observation in STS tissue, we tested the above-mentioned cell lines with regard to stress-induction of *ASSP2κ* when changing cell culture temperature conditions.

Cells were incubated at 37 °C or room temperature (RT) overnight and *ASSP2κ* mRNA expression levels were assessed by isoform-specific qRT-PCR. Indeed, all tested sarcoma cell lines cultured at RT displayed significantly higher *ASPP2κ* levels than the cell strains incubated at 37 °C (Fig. 1B).

### ASPP2κ is expressed in native rhabdomyosarcoma tissue

The cell line experiments revealed that the highest expression levels of *ASPP2κ* were detected in rhabdomyosarcoma tissue. We therefore further concentrated on this histologic subtype to determine ASPP2κ expression levels in patient-derived tumors. Isoform-specific qRT-PCR was used to assess *ASPP2κ* mRNA expression in snap-frozen rhabdomyosarcoma samples from 15 consented patients. Snap-frozen biopsies from surrounding tumor-free tissue were used as a baseline expression control.

Even though we noted patient-to-patient variability of *ASPP2κ* expression levels, we found that median expression of *ASPP2κ* was significantly higher in rhabdomyosarcoma in comparison to a pool of tumor-free tissue (Fig. 1C). Importantly, analysis of tumor tissue versus adjacent, microscopically tumor-free tissue in individual patient samples, confirmd tumor-specific increase of *ASPP2κ* in sarcoma cells (Fig. 1D).

**Fig. 2:**
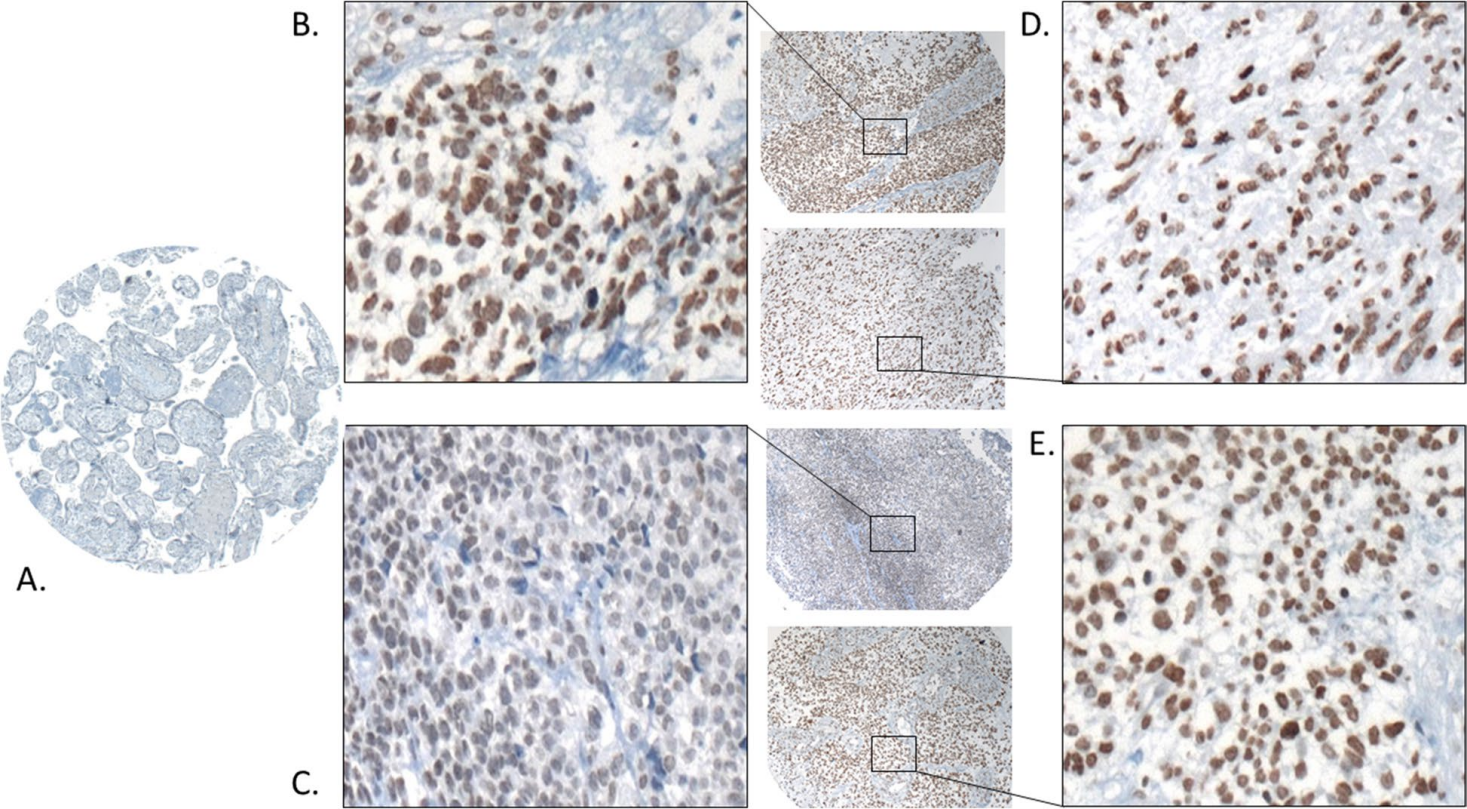
FFPE rhabdomyosarcoma patient samples stained for ASPP2κ protein expression, using an isoform-specific antibody (ab#5385 [13]) (**B**-**E**), (**A**) Normal placenta tissue served as a negative control. (10 × magnification, zoom 100x)

Tumor-specificity of *ASPP2κ* was next confirmed on the protein level using isoform-specific ASPP2κ antibodies detecting the genuine truncated protein isoforms [13]. A panel of eleven formaldehyde-fixed paraffin-embedded (FFPE) native patient-derived rhabdomyosarcoma samples was analysed – confirming significant overexpression of ASPP2κ (7/11) in the tumor tissue. Mesenchymal placenta tissue served as a basal expression control (Fig. 2).

Taken together, these experiments provide the first evidence that ASPP2κ is frequently overexpressed in human rhabdomyosarcomas.

### ASPP2κ-interference enhances induction of apoptosis

To investigate whether or not ASPP2κ*-*affects the sensitivity of rhabdomyosarcoma cells towards chemotherapy, isoform-specific *ASPP2κ*-interference RD and ssRMS-based models were established using a lentiviral transduction approach (see the *methods* section for further details).

Anthracyclines are a hallmark therapeutic in the treatment of STS. We therefore used doxorubicin to treat shASPP2κ.RD and shASPP2κ.ssRMS cell strains and determined the potential to induce apoptosis in comparison to the respective EV cell strains. Cells were treated in dose-dilution series for 48 h and the proportion of apoptotic cells was determined using an annexin V-based flow cytometry assay.

**Fig. 3:**
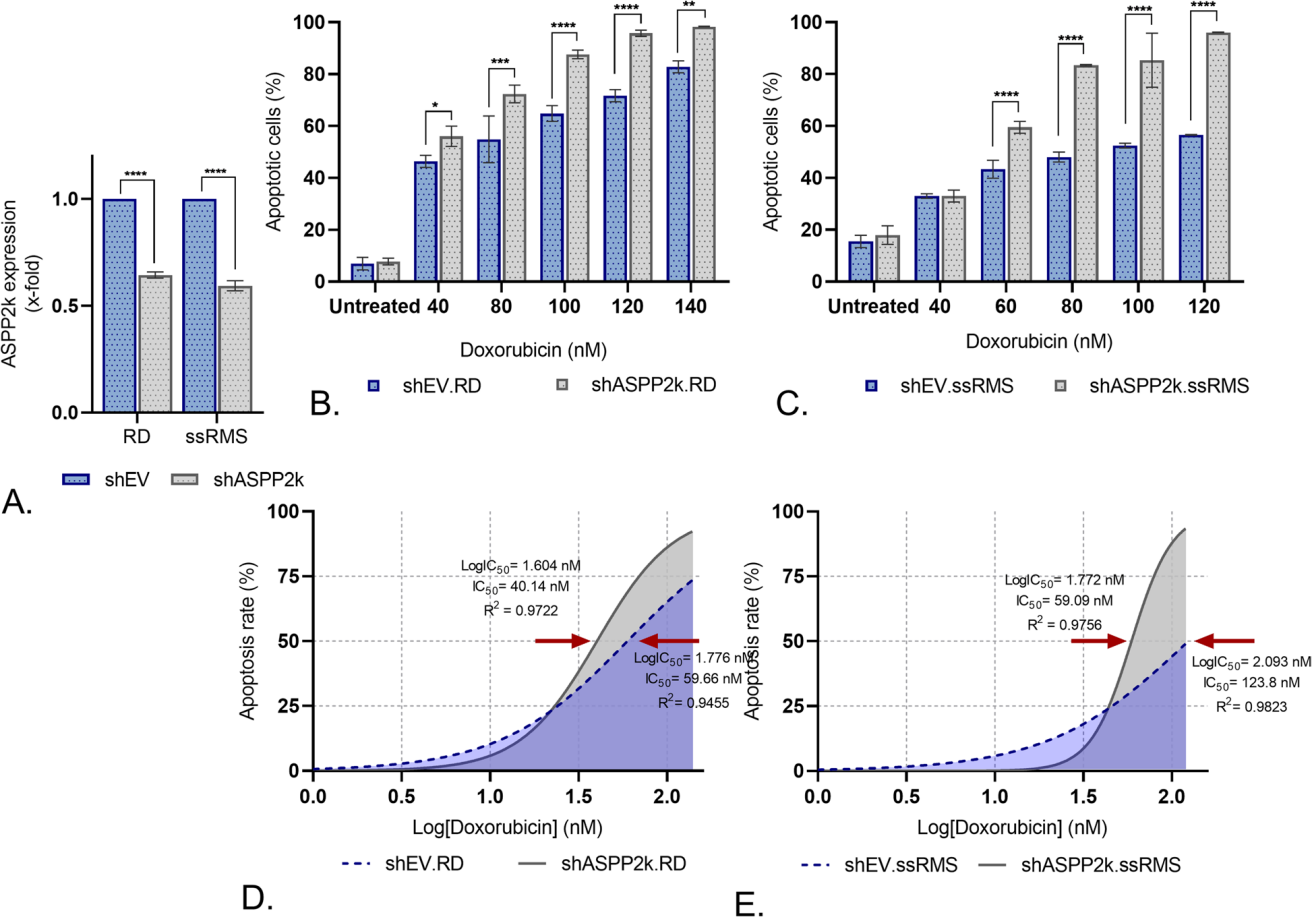
**A** Verification of specific hairpin-induced ASPP2κ-interference using isoform-specific qRT-PCR. EV, empty vector. Statistical test: unpaired t-test. **B**, **C** Induction of apoptosis upon treatment with doxorubicin in ASPP2κ-interferenced RD and ssRMS cells, when compared to the respective EV control stains. Statistical test: two-way ANOVA. ****p < 0.0001, ***p < 0.001, ** *p* < 0.01, *p < 0.05. **D**, **E** Computed logIC_50_ and IC_50_ values of shASPP2κ or shEV.RD, resp. ssRMS, cell strains in response to doxorubicin

Notably, attenuation of *ASPP2κ* significantly increased the apoptosis rate upon exposure to doxorubicin chemotherapy in both tested cell lines (Fig. 3B, C). Specifically, the IC_50_ dropped by approximately 33% in shASPP2κ.RD in comparison to shEV.RD, while for shASPP2κ.ssRMS cells the IC_50_ dropped by 52% when compared to the EV control strain (Fig. 3D, E). This observation argues for a strong dominant-negative effect of ASPP2κ – even more as interference efficiency in both models was only ~ 40% (Fig. 3A).

### *ASPP2κ*-interference attenuates cellular proliferation rates

Although ASPP2 was originally described as an apoptotic modulator, increasing evidence suggests additional biological functions in cellular growth and movement [16, 17]. We therefore aimed to assess whether ASPP2κ affects cellular proliferation rates in our *ASPP2κ*-interferenced rhabdomyosarcoma models.

**Fig. 4:**
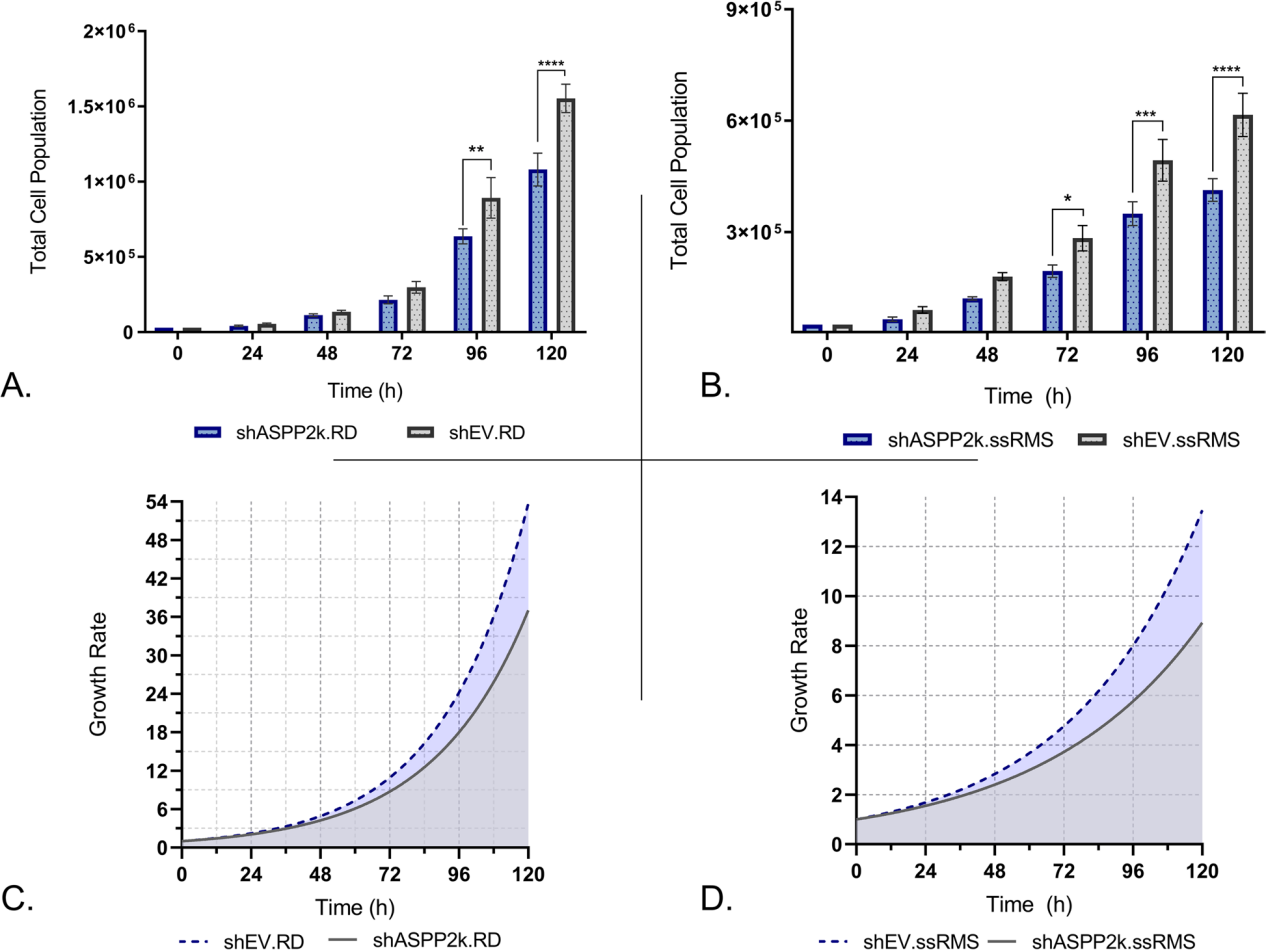
**A**, **B** Proliferation rates of ASPP2κ-interferenced RD and ssRMS cell strains vs empty vector (EV) controls. Statistical test: two-way ANOVA. **** *p* < 0.0001, *** *p* < 0.001, ** *p* < 0.01, * *p* < 0.05 (**C**), (**D**) Computed growth rates in dependence of ASPP2κ expression. The assay was performed in 3 × technical triplicates

All cell strains were seeded at equal cell numbers per well; culture and growth capacity were followed over time. We found significant attenuation of cellular proliferation rates in the *ASPP2κ-*interferenced cell strains in comparison to the EV controls in both rhabdomyosarcoma models (Fig. 4A, B).

The exponential growth equation was calculated for each cell line, showing that cell doubling times of the *ASPP2κ*-interferenced cell strains were significantly decreased in comparison to the EV controls. Specifically, the doubling time of *ASPP2κ*-interferenced cells increased by 3 (RD) and 6 (ssRMS) hours, a difference that led to an average decrease of 29% (RD) and 36% (ssRMS) of the growth rate during a five-day follow-up period (Fig. 4C, D).

### *ASPP2*-interference attenuates migration of rhabdomyosarcoma cells

**Fig. 5:**
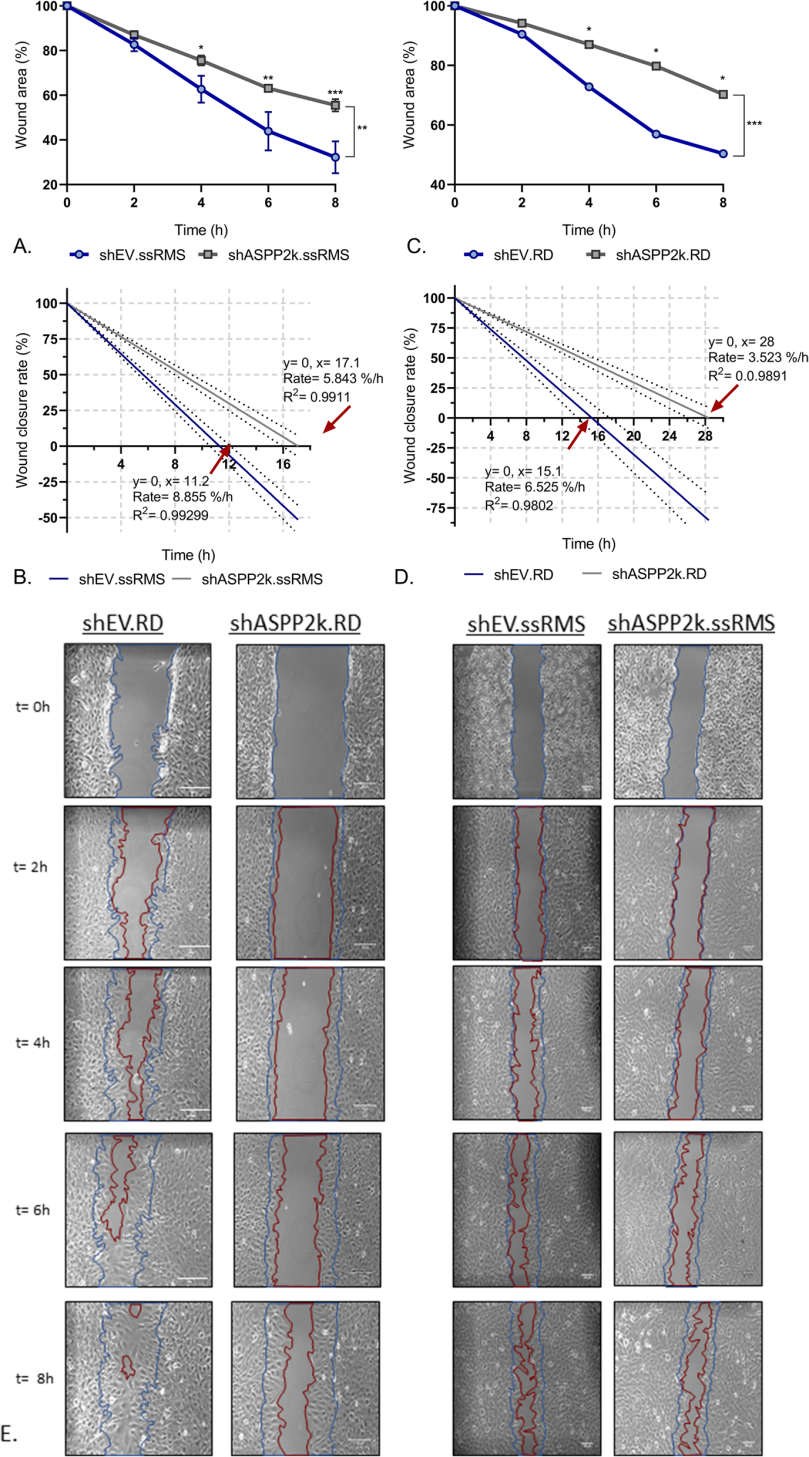
ASPP2κ-interference inhibits cell migration in rhabdomyosarcoma cells. **A**, **C** Quantitative analysis of wound-healing rates in rhabdomyosarcoma cells. Statistical test: two-way ANOVA. **B**, **D**. Linear regression graph of the wound-healing speed. EV, empty vector. **** *p* < 0.0001, *** *p* < 0.001, ** *p* < 0.01, * *p* < 0.05. **E** Illustration of representative wound healing experiments for the shASPP2κ*-*RD and ssRMS cell lines for five time points (t = 0-8 h). Graphical display of wound healing computed by ImageJ. Blue curve, overlay of wound margin at the start of experiment; red curve, wound closure at a given time point; experiments were performed in technical triplicates

Microarray mRNA experiments on ASPP2 ± MEF revealed multiple functions of ASPP2, including cellular movement [16, 17]. To explore whether ASPP2κ*-*could promote tumorigenesis via cellular movement, we utilized a wound-healing assay (methodology described in detail in the *methods* section) and herein confirm a role of ASPP2κ in controlling cellular migration (Fig. 5A-E). Importantly, ASPP2κ-interference attenuates cell motility as demonstrated by a decreased wound closure time when compared to the respective EV control strains (Fig. 5A, C and E). The wound-healing rate was calculated from the linear regression equation (Fig. 5B, D) as approximately 8.9% per hour for shEV.ssRMS cells, in comparison to 5.8% per hour for shASPP2κ. ssRMS (Fig. 5B). For the RD cell line, the wound closure rate was calculated as 6.5% and 3.5% per hour for the control and *ASPP2κ*-interferenced cells, respectively (Fig. 5D). As a consequence, the total wound closure time was estimated at 11 h (ssRMS) and 15 h (RD) for the EV control strains, whereas the respective *ASPP2κ-*interferenced cell strains achieved full wound healing after 17 h (ssRMS) and 28 h (RD) (Fig. 5B, D) (i.e., attenuation by 34% in ssRMS, resp. 46% in the RD cell line model). This data demonstrates that ASPP2κ promotes cellular migration, consistent with a role as a tumor-promoting, oncogenic driver.

## Discussion

Rhabdomyosarcoma (RMS) are the most common pediatric and juvenile STS involving around 5% of all childhood tumors, while being rare in adults. According to WHO, RMS subtypes are classified as embryonal rhabdomyosarcoma (ERMS, corresponding to the RD cell line), which comprises the largest group of soft tissue malignancies in children and adolescents, alveolar rhabdomyosarcoma (ARMS), pleomorphic rhabdomyosarcoma (PRMS), and spindle cell/sclerosing rhabdomyosarcoma (ssRMS) [18]. Prognosis thereby depends on the RMS subtype: with superior prognosis of botryoid embryonal subtype RMS and spindle cell or leiomyomatous RMS, followed by embryonal RMS and alveolar RMS.

Standard treatment includes chemotherapy, surgery, and/or radiation therapy. Despite improved cure rates with the advent of anthracycline-based combination chemotherapy, patients with advanced disease and especially with relapsing disease still face high mortality rates. A better understanding of the cellular and molecular complexity of human sarcomas remains crucial to unveil the molecular basis of sarcomagenesis and to design novel therapeutics to improve clinical outcome.

The apoptosis-stimulating protein of TP53-2 (ASPP2), encoded by *TP53BP2*, is an independent tumor suppressor, enhancing damage-induced apoptosis at least in part through a TP53-mediated pathway [6]. Dysfunctional expression of ASPP2 has been observed in different tumor entities and downregulation of ASPP2 has been shown to go along with poorer prognosis [6, 8, 9, 12].

Still, the underlying mechanisms of action are poorly understood and the identification of truncated ASPP2 isoforms have added an additional level of complexity to the issue [6, 13, 19]. In this context, we recently identified a dominant-negative splicing isoform, *ASPP2κ,* with a high prevalence in acute leukemia, contributing to leukemogenesis and therapy resistance [13]. ASPP2κ lacks most of the C-terminus of ASPP2, containing the p53 binding domain [13], and failure of proper ASPP2/p53 interaction is predicted to disrupt the apoptotic function of p53 [6]. This is of utmost interest as many major *TP53* mutations lead to loss of the ASPP2 binding sites, which again results in impaired induction of apoptosis [14].

Our recent *ASPP2* +/- mouse model has demonstrated that hemizygous mice have an increased tumor formation rate compared to the *ASPP2* wt controls. Interestingly, we observed a high rate of STS in these models [10], arguing for a special biological role of ASPP2 in these tumor entities. We therefore aimed to evaluate whether ASPP2κ is expressed and plays a functional role in STS:

Indeed, a screen of a number of sarcoma cell lines revealed frequent and stress-inducible expression of *ASPP2κ* in STS – whereas expression levels varied widely. Two rhabdomyosarcoma cell models, namely the RD embryonal and the ssRMS spindle cell/sclerosing rhabdomyosarcoma cell line displayed the highest expression levels – so we next concentrated on studying ASPP2κ expression in native rhabdomyosarcoma tissue. In a pool of 15 patients, the median *ASPP2κ* expression level was significantly elevated when compared to a healthy tissue control pool. However, patient-to-patient variability is meaningful – and future studies will need to address this observation to define sub-entities according to expression levels, especially in the context of therapy resistance and risk of metastasis as discussed later.

Of note, even in tumors with relatively low *ASPP2κ* levels, analysis of adjacent surrounding healthy tissue from the same rhabdomyosarcoma sample revealed that *ASPP2κ* is specifically expressed in the tumor cells. This observation was further confirmed on the protein level using an immunohistochemistry approach to detect expression and distribution of ASPP2κ in native RMS tumor tissue with custom-made isoform specific anti-ASPP2κ antibodies [13].

Intriguingly, in our initial expression screen, ASPP2κ was not uniquely found in RMS sub-entities alone, but was also detected in other STS entities, such as leiomyosarcoma and liposarcoma (Supplemental figure S1). This implicates that alternative splicing of ASPP2 is a frequent event in sarcoma, which may have functional consequences such as promoting sarcomagenesis.

To determine the functional significance of ASPP2κ expression in rhabdomyosarcoma, we employed an isoform-specific *ASPP2κ*-interference approach to stably attenuate *ASPP2κ* in two RMS cell lines (RD, ssRMS). Consistent with our prior data in leukemia models [13], we found that modulating expression of ASPP2κ significantly alters the response of sarcoma cells to chemotherapy-induced apoptosis. Precisely, ASPP2κ-interference rendered RMS cells more susceptible towards anthracycline-induced apoptosis. These results provide additional significance to our observation showing an increase of ASPP2κ expression level upon stress-induction (Fig. 1B), as it argues that ASPP2κ may be an important mechanism for chemotherapy resistance.

Although ASPP2 was originally described as an apoptotic modulator, it harbors additional biological functions as implicated for example by its interaction with RAS [16, 17]. Further, mRNA microarray network analyses using mouse embryonic fibroblasts (MEF) from our mouse ASPP2  knock-out model have suggested a dominant role for ASPP2 in cellular growth and movement [16]. Indeed, employing our isoform-specific *ASPP2κ* silencing approach, we demonstrate that cellular proliferation rates depend on *ASPP2κ* expression and isoform-specific silencing of ASPP2κ results in significant deceleration of growth rates in both RMS cell models tested. Tantalizingly, further studies confirm significant attenuation of migration and wound closure capacities in ASPP2κ silenced cell models. This observation is exciting, pointing to a new functional role of *ASPP2κ.*

Together, given the constantly expanding repertoire of ASPP2 pathway functions, our current findings expand this notion with ever increasing complexity, by demonstrating the role of *ASPP2κ* in controlling cellular proliferation, movement and migration in rhabdomyosarcoma models – suggesting a functional role of ASPP2κ in promoting sarcomagenesis.

Our observations are not limited to RMS: Additional work on liposarcoma (provided as supplemental figure S1, including additional information about expression profiles in native patient tissue as well as functional data in ASPP2κi liposarcoma cell models) underlines this notion and suggests a general role of ASPP2κ in STS, warranting further systematic exploration of ASPP2κ in other sarcoma-subtypes as well.

Together, these observations argue for a central role of *ASPP2κ* in sarcoma biology, response to therapy, proliferation and potential to metastasize. Although the precise molecular mechanisms for *ASPP2κ* in sarcoma-genesis remain to be elucidated in detail, our model systems provide a robust experimental platform that will permit further exploration of these important pathways. This includes exploration of ASPP2κ as a potential new biomarker for response to therapy as well as a potential new target for novel therapeutics, targeting ASPP2κ directly or other deregulated pathways in sarcoma, which may intersect with ASPP2 and ASPP2κ pathways.

## Conclusions

Taken together, we demonstrate that oncogenic ASPP2κ is frequently expressed in STS and promotes cellular proliferation and migration as well as attenuated induction of chemotherapy-induced apoptosis: all hallmarks of cancer [20]. Given the significant clinical challenges of treating sarcoma in patients, our findings provide significant new insights into the understanding of the complex regulation and function of ASPP2 in STS.

Our data provide a strong rationale for future studies to evaluate ASPP2κ expression in different sarcoma entities, as it may serve as a clinically valuable prognostic tool to predict the risk of metastasis or response to therapy. In the future, ASPP2κ may be therapeutically targetable to sensitize tumor cells towards radiation or chemotherapy with the goal to further improve patient outcomes.

## Supplementary Information

**Additional file 1:** **Supplemental Table 1. **Rhabdomyosarcoma patient characteristics. **Supplemental Table 2. **Liposarcoma patient characteristics. **Supplemental Figure S1.** (A) *ASPP2κ-*specific qRT-PCR assay to determine relative mRNA expression levels in native liposarcoma patient tissue (*n*=15). A sample pool (*n*=7) of tumor-free tissue derived from the same patients serves as the control. GAPDH serves as a housekeeping gene. Each patient sample was measured in technical triplicates. Statistical test: one-sample and unpaired t-test (B) *ASPP2κ*-specific qRT-PCR assay to determine relative mRNA expression levels (GAPDH serves as housekeeping gene) between tumor and healthy tissue from the same individual (*n*=4) Statistical test: two-way ANOVA. (C) Verification of hairpin induced specific *ASPP2κ-*interference using isoform-specific qRT-PCR. EV, empty vector in liposarcoma cell line SW872. Statistical test: unpaired t-test (D) Growth rates in dependence of ASPP2κ in SW872 cell line (*n*=9). (E) Induction of apoptosis upon treatment with doxorubicin in ASPP2*κ*-silenced SW872 cells compared to the respective EV control strains (*n*=3). Statistical test: two-way ANOVA ****p< 0.0001, ***p<0.001, ** *p* < 0.01, *p < 0.05. 

## Data Availability

All data generated or analyzed during this study are included in this article and its supplementary information files.

## Abbreviations

7-AAD: 7-Amino actinomycin
ANOVA: Analysis of variance
ARMS: Alveolar rhabdomyosarcoma
ASPP1/2: Apoptosis stimulating protein of p53 1 or 2
CMV: Cytomegalovirus
Cp: Crossing point
DAB: 3,3’-Diaminobenzidine
DMEM: Dulbecco’s modified eagle’s medium
DMSO: Dimethyl sulfoxide
DNA: Deoxyribonucleic Acid
ERMS: Embryonal Rhabdomyosarcoma
EV: Empty vector
FACS: Fluorescence activated cell sorting
FBS: Fetal bovine serum
FFPE: Formalin-fixed paraffin-embedded
GAPDH: Glyceraldehyde 3-phosphate dehydrogenase
GFP: Green fluorescent protein
iASPP: Inhibitor of ASPP protein (aka RelA-associated inhibitor)
IC50: Half-maximal inhibitory concentration
MEF: Mouse embryonic fibroblasts
MEM: Minimum Essential Medium
PBS: Phosphate buffer saline
PDB: Protein data bank
PRMS: Pleomorphic rhabdomyosarcoma
qRT-PCR: Quantitative reverse transcription polymerase chain reaction
RAS: GTPase Rat-sarcoma proteins
RMS: Rhabdomyosarcoma cell line
RPMI: Roswell park memorial institute medium
RT: Room temperature
SH3: SRC homology 3
shRNA: Short hairpin ribonucleic acid
siRNA: Silencing ribonucleic acid
ssRMS: Spindle cell/sclerosing rhabdomyosarcoma cell line
STS: Soft tissue sarcoma
SV40: Simian virus 40
TP53: Tumor protein 53
TP53BP2: Tumor protein 53 binding protein 2

## References

[1] Gustafson P (1994). Soft tissue sarcoma. Epidemiology and prognosis in 508 patients. Acta Orthop Scand Suppl.

[2] Parker SL, Tong T, Bolden S, Wingo PA (1996). Cancer statistics, 1996. CA Cancer J Clin.

[3] Fletcher CD, Hogendoorn P, Mertens F, Bridge J. WHO Classification of Tumours of Soft Tissue and Bone. 4th ed. Lyon: IARC Press; 2013.

[4] Trigiante G (2006). ASPPs and cancer. Nature reviews.

[5] Bergamaschi D, Samuels Y, O'Neil NJ, Trigiante G, Crook T, Hsieh JK (2003). iASPP oncoprotein is a key inhibitor of p53 conserved from worm to human. Nat Genet.

[6] Samuels-Lev Y, Bergamaschi D, O’Connor DJ, Trigiante G, Hsieh JK, Zhong S (2001). ASPP Proteins Specifically Stimulate the Apoptotic Function of p53. Mol Cell.

[7] Bergamaschi D, Samuels Y, Jin B, Duraisingham S, Crook TXL (2004). ASPP1 and ASPP2: Common Activators of p53 Family Members. Mol Cell Biol.

[8] Lossos IS, Natkunam Y, Levy R (2002). CDL, Apoptosis Stimulating Protein of p53 (ASPP2) Expression Differs in Diffuse Large B-cell and Follicular Center Lymphoma: Correlation with Clinical Outcome. Leuk Lymphoma.

[9] Schittenhelm MM, Illing B, Akmut F, Rasp KH, Blumenstock G, Döhner K (2013). Attenuated Expression of Apoptosis Stimulating Protein of p53–2 (ASPP2) in Human Acute Leukemia Is Associated with Therapy Failure. PLOS One.

[10] Kampa KM, Acoba JD, Chena D, Gaya J, Leea H, Beemera K (2009). Apoptosis-stimulating protein of p53 (ASPP2) heterozygous mice are tumor-prone and have attenuated cellular damage–response thresholds. PNAS.

[11] Vives V, Su J, Zhong S, Ratnayaka I, Slee E, Goldin R (2006). ASPP2 is a haploinsufficient tumor suppressor that cooperates with p53 to suppress tumor growth. Genes Dev.

[12] Wang Y, Godin-Heymann N, Dan Wang X, Bergamaschi D, Llanos S, Lu X (2013). ASPP1 and ASPP2 bind active RAS, potentiate RAS signalling and enhance p53 activity in cancer cells. Cell Death Differ.

[13] Schittenhelm MM, Walter B, Tsintari V, Federmann B, Bajrami-Saipi M, Akmut F (2019). Alternative splicing of the tumor suppressor ASPP2 results in a stress-inducible, oncogenic isoform prevalent in acute leukemia. EBioMedicine.

[14] Gorina S, Pavletich NP (1996). Structure of the p53 tumor suppressor bound to the ankyrin and SH3 domains of 53BP2. Science.

[15] Gebäck T, Schulz MMP, Koumoutsakos P, Detnar M (2009). TScratch: a novel and simple software tool for automated analysis of monolayer wound healing assays. BioTechniques.

[16] Kampa KM, Bonin M, Lopez CD (2009). New insights into the expanding complexity of the tumor suppressor ASPP2. Cell Cycle.

[17] Wang Z, Liu Y, Takahashi M, Van Hook K, Kampa-Schittenhelm KM, Sheppard BC (2013). N terminus of ASPP2 binds to Ras and enhances Ras/Raf/MEK/ERK activation to promote oncogene-induced senescence. PNAS.

[18] Sun X, Guo W, Shen JK, Mankin HJ, Hornicek FJ (2015). Duan ZRhabdomyosarcoma: Advances in Molecular and Cellular Biology. Sarcoma.

[19] Van Hook K, Wang Z, Chen D, Nold C, Zhu Z, Anur P (2017). DeltaN-ASPP2, a novel isoform of the ASPP2 tumor suppressor, promotes cellular survival. Biochem Biophys Res Commun.

[20] Hanahan D, Weinberg RA (2011). Hallmarks of Cancer: The Next Generation. Cell.

