# Supplementary material for: Alternative splicing of Apoptosis Stimulating Protein of TP53-2 (ASPP2) results in an oncogenic isoform promoting migration and therapy resistance in soft tissue sarcoma (STS)
Source: BMC Cancer. 2022 Jul 2;22:725. doi: 10.1186/s12885-022-09726-7 (PMC9250729; doi:10.1186/s12885-022-09726-7)
Supplement: Supplementary file 1 — Additional file 1: Supplemental Table 1. Rhabdomyosarcoma patient characteristics. Supplemental Table 2. Liposarcoma patient characteristics. Supplemental Figure S1. (A) ASPP2κ-specific qRT-PCR assay to determine relative mRNA expression levels in native liposarcoma patient tissue (n=15). A sample pool (n=7) of tumor-free tissue derived from the same patients serves as the control. GAPDH serves as a housekeeping gene. Each patient sample was measured in technical triplicates. Statistical test: one-sample and unpaired t-test (B) ASPP2κ-specific qRT-PCR assay to determine relative mRNA expression levels (GAPDH serves as housekeeping gene) between tumor and healthy tissue from the same individual (n=4) Statistical test: two-way ANOVA. (C) Verification of hairpin induced specific ASPP2κ-interference using isoform-specific qRT-PCR. EV, empty vector in liposarcoma cell line SW872. Statistical test: unpaired t-test (D) Growth rates in dependence of ASPP2κ in SW872 cell line (n=9). (E) Induction of apoptosis upon treatment with doxorubicin in ASPP2κ-silenced SW872 cells compared to the respective EV control strains (n=3). Statistical test: two-way ANOVA ****p< 0.0001, ***p<0.001, ** p < 0.01, *p < 0.05. [file 12885_2022_9726_MOESM1_ESM.pdf]

Supplemental Table 1: Rhabdomyosarcoma patient characteristics

Patient Nr.	Age	Sex	Diagnosis
R1	4.5	F	relapse of RMS with poor differentiation
R2	4.5	M	embryonic RMS
R3	1.5	M	embryonic RMS
R4	3.5	M	embryonic RMS
R5	2.5	F	botryoid RMS
R6	4.5	M	nodular infiltrates of an RMS
R7	4.5	M	intra-abdominal RMS
R8			no further data available
R9	60	M	RMS dorsal upper arm
R10	1.5	F	embryonic RMS
R11	4	F	no further data available
R12			no further data available
R13	2	M	embryonic RMS
R14	1	M	embryonic RMS
R15	2.5	F	embryonic RMS
R16	17	F	embryonic RMS

Supplemental Table 2: Liposarcoma patient characteristics

Patient Nr.	Age	Sex	Diagnosis
L1	77	M	liposarcoma of the kidney
L2	60	M	highly differentiated liposarcoma
L3	70	M	thoracic liposarcoma
L4	61	F	relapse of retroperitoneal liposarcoma
L5	63	M	well-differentiated liposarcoma
L5			no further data available
L6	66	M	atypical lipoma/highly differentiated liposarcoma
L7	54	F	atypical lipoma/highly differentiated liposarcoma
L8	58	F	round cell myxoid liposarcoma
L9	58	M	spindle cell high-grade sarcoma, G3
L10	47	M	retroperitoneal liposarcoma
L11	47	F	myxoid liposarcoma
L12			no further data available
L13	40	M	differentiated liposarcoma
L14	47	M	recurrent liposarcoma
L15	73	M	pleomorphic liposarcoma
L16	71	F	retroperitoneal liposarcoma

Supplemental Figure S1

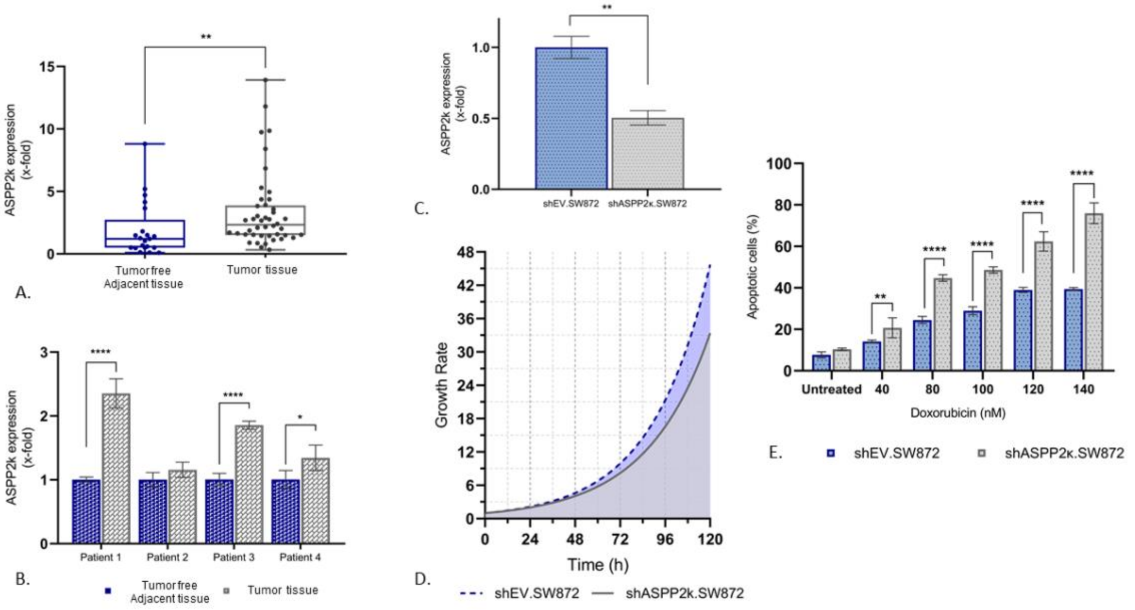

(A) *ASPP2κ*-specific qRT-PCR assay to determine relative mRNA expression levels in native liposarcoma patient tissue (n=15). A sample pool (n=7) of tumor-free tissue derived from the same patients serves as the control. GAPDH serves as a housekeeping gene. Each patient sample was measured in technical triplicates. Statistical test: one-sample and unpaired t-test (B) *ASPP2κ*-specific qRT-PCR assay to determine relative mRNA expression levels (GAPDH serves as housekeeping gene) between tumor and healthy tissue from the same individual (n=4) Statistical test: two-way ANOVA. (C) Verification of hairpin-induced specific *ASPP2κ*-interference using isoform-specific qRT-PCR. EV, empty vector in liposarcoma cell line SW872. Statistical test: unpaired t-test (D) Growth rates in dependence of ASPP2κ in SW872 cell line (n=9). (E) Induction of apoptosis upon treatment with doxorubicin in ASPP2*κ*-silenced SW872 cells compared to the respective EV control strains (n=3). Statistical test: two-way ANOVA ****$p < 0.0001$, ***$p<0.001$, ** $p < 0.01$, *$p < 0.05$
